# Site-specific N-glycoproteomics reveals alteration of N-glycan structures and heterogeneity to enhance thermally induced gelation characteristics of egg white proteins

**DOI:** 10.1016/j.fochx.2026.104473

**Published:** 2026-09-20

**Authors:** Guang Li, Jiao Wang, Pengyun Gong, Yongguo Jin, Bingkun Zhang, Yuming Guo, Zengpeng Lv

**Affiliations:** a State Key Laboratory of Animal Nutrition and Feeding, China Agricultural University, Beijing 100193, China; b Fetal Cardiopaternal-Maternal Medicine Center, Beijing Anzhen Hospital, Capital Medical University, Beijing 100029, China; c National Research and Development Center for Egg Processing, College of Food Science and Technology, Huazhong Agricultural University, Wuhan, Hubei 430070, PR China; d Sanya Institute, China Agricultural University, Sanya 572025, China; e China Agricultural University-Sichuan Advanced Agricultural & Industrial Institute, Chengdu 610046, China

**Keywords:** Egg white protein, Site-specific N-glycan, Glycan structure, N-glycoproteomics, Thermally induced gel

## Abstract

N-glycans are complex carbohydrates crucial for the structural integrity and functionality of proteins. Here, we present a high-resolution egg white protein N-glycoproteomic atlas that systematically unveils the microheterogeneity across N-glycosites, glycoproteins, and co-occurring N-glycans. Notably, hawthorn leaf flavonoids (HF) markedly reduced undecorated and high-mannose N-glycans while increasing paucimannose and sialylated-only N-glycans, and these changes strongly correlated with elevated protein stability. Site-specific N-glycan comparative analysis further revealed that HF increased 15 N-glycans at four glycoproteins (such as Hex_9_GlcNAc_2_ in ovotransferrin N492) and reduced 3 N-glycans at three glycoproteins (such as Hex_3_HexNAc_6_ in mucin-5B N69), promoting the formation of more ionic bonds, hydrophobic interactions, and hydrogen bonds, thereby converting the secondary structure from random coil to α-helix and β-sheet and ultimately forming a more tightly ordered egg white gel. Overall, this study provides a novel perspective on improving gel properties by modulating the structure and site-specific N-glycosylation of egg white proteins (EWPs).

## Highlights

• Site-specific N-glycoproteomic landscape of EWPs was elucidated.
• Revealed heterogeneity: glycosites, glycoproteins, glycoforms, and co-occurring glycans.
• N-glycans of OVM, Mucin-5B, and OVT participated in regulating the quality of EWP gel.
• EWP gels with HF exhibited denser and more ordered structures.

## 1 Introduction

Egg white is a premium dietary protein source with a balanced amino acid profile, accounting for 10%-13% of total egg weight. Its primary proteins confer outstanding nutritional value and excellent functional properties (Liu et al., 2021). Leveraging its excellent gelling, foaming, and emulsifying capacities, egg white protein is extensively utilized across various food applications, including meat products, baked goods, and frozen desserts (Alavi et al., 2020). Among these, gelling properties are one of the core indicators for evaluating the processing quality of eggs, directly determining their application value in meat products, baked goods, and other applications (Zhang, Mei, et al., 2024). Their formation is intimately related to protein molecular conformation, aggregation behavior, and intermolecular forces (Liu et al., 2024; Zhang et al., 2022). However, the internal quality and gel properties of egg white significantly decline with the aging of laying hens, manifesting as reduced gel strength, decreased water holding capacity (WHC), and deteriorated microstructure, which severely limit the processing value and economic benefits of egg white (Li et al., 2023). Therefore, the development of safe and effective nutritional strategies to improve egg white gel characteristics during the late laying period has become an urgent and critical issue in the fields of egg science and poultry production.

Egg white gel properties highly depend on the composition, structural conformation, and post-translational modifications of its constituent proteins (Li, Chen, et al., 2025; Li, Guan, et al., 2025; Liu et al., 2021). Changes in the abundance of ovalbumin and mucin-5B during the aging of laying hens are accompanied by alterations in the secondary structure and intermolecular interactions of EWPs, ultimately leading to the formation of rough, porous, and structurally weak gel networks (Li et al., 2023; Li, Chen, et al., 2025). Furthermore, N-glycosylation, as one of the most important post-translational modifications of proteins, plays a key role in regulating the functional characteristics of EWPs by impacting the folding, stability, and intermolecular interactions of glycoproteins (Dai et al., 2025; Qi et al., 2024; Zhang et al., 2026). Covalent cross-linking of N-glycan structures was shown to significantly impact thermal aggregation behavior in EWPs during heat processing (Li, Guan, et al., 2025; Liu et al., 2021). Notably, the highly N-glycosylated protein, ovomucin, plays a pivotal role in the nascent stages of thermogel formation, a process largely governed by the structural heterogeneity conferred by glycan attachments (Wang et al., 2022). Recently, it has also been found that differences in site-specific N-glycan structures can directly alter the thermal aggregation behavior of critical proteins, including ovalbumin and mucin-5B, whereas changes in protein glycosylation induced by nutritional interventions may serve as a central mechanism regulating the functional properties of EWPs (Li, Guan, et al., 2025; Liu et al., 2021; Zhou et al., 2025).

To date, the systematic investigation of N-glycan structural diversity from glycoproteomics data to construct an N-glycoproteomic atlas of EWPs has rarely been conducted. Only a limited number of studies have reported 97 and 487 unique intact N-glycopeptides, 19 and 40 unique N-glycoproteins, as well as 55 and 104 distinct N-glycan compositions in EWPs, respectively (Cavallero et al., 2020; Li, Guan, et al., 2025). However, previous glycoproteomics studies primarily focused on the relationship between the number of N-glycosites and egg white function, while neglecting the impact of the structures and heterogeneity of N-glycans at N-glycosites on egg white function (Geng et al., 2017; Liu et al., 2021). In addition, N-glycosylation has been reported to increase protein solubility through the introduction of hydrophilic glycan moieties and to improve emulsifying properties by virtue of the spatial conformation of glycan chains (Zhang et al., 2019). Moreover, variations in glycosites can lead to different positions and lengths of the glycan chains, thereby inducing alterations in the three-dimensional conformation of the protein (An et al., 2009). Thus, we integrated site-specific N-glycoproteomics data with egg white gel property data, presenting a high-resolution egg white protein N-glycoproteomic atlas, revealing how distinct glycan structures and site-specific glycosylation improve gel characteristics of EWPs in late-laying hens. Collectively, the study expands the scope of glycoproteomics and provides glycobiological insights into optimizing egg white gel properties applied in food manufacturing.

## 2 Materials and methods

### 2.1 Collection and preparation of egg white samples

This study collected fresh eggs from 78-week-old “Jing Tint 6” laying hens housed at the China Agricultural University Experimental Base (Zhuozhou, Hebei, China). The hens were fed either a basal diet (control) or a basal diet containing 120 mg/kg HF from 66 to 78 weeks of age. Fresh eggs were carefully cracked by hand to separate the egg white from the egg yolk and chalaza. An appropriate amount of egg white was homogenized using a magnetic stirrer and transferred to a culture dish, sealed with parafilm, and incubated in a water bath at 90 °C for 30 min to obtain egg white protein gels. Following heat treatment, the gel was immediately placed in an ice-water bath to cool, followed by overnight equilibration at 4 °C for subsequent analysis of gel properties.

### 2.2 Egg white thermogel properties measurement

#### 2.2.1 Texture characteristics and WHC

Gel samples were cubed into uniform 1.5 cm × 1.5 cm cubes, equilibrated under ambient conditions, and then analyzed for texture profiles using a texture analyzer (TA. TOUCH, BosinTech, China) equipped with a T/36 cylindrical probe (Liu et al., 2020). The texture analyzer was operated with the following settings: the pre-test, test, and post-test speeds were set to 8.00, 1.00, and 8.00 mm/s, respectively. The sample was compressed to 50% of its original height, and the trigger force was fixed at 5 g. The hardness, springiness, chewiness, and gumminess of egg white gels were all examined. Texture Exponent software was used to collect data from the test curves. Each sample was tested in triplicate.

The WHC of thermally induced gels was analyzed using a previously reported centrifugation method (Zhang, Mei, et al., 2024). Specifically, the initial thermally induced gels were precisely weighed (this weight was defined as m1) and then subjected to 30 min of centrifugation at 10,000 ×*g* at 4 °C in 50 mL tubes. After centrifugation, the gels were reweighed, and this weight was recorded as m2. The following equation was used to calculate the WHC (%) (1):

*WHC* (%) = *m1/m2* × 100% (1)

#### 2.2.2 Low-field nuclear magnetic resonance (LF-NMR) and magnetic resonance imaging (MRI)

The measurement of water distribution in egg white gels was performed on an LF-NMR (NMI20-015 V-I, Niumag Electric Co., Ltd., Suzhou, China), following a previously described approach (Wu et al., 2024). In brief, about 1 g of the cylindrical thermal gel was placed in an NMR glass tube (25 mm in diameter), inserted into the NMR probe, and then the transverse relaxation time (T_2_) was determined using the Carr-Purcell-Meiboom-Gill (CPMG) sequence. The parameters were set as follows: echo time was 0.35 ms, the number of echoes was 12,000, the cumulative number of scan repetitions was 16, and the waiting time was 5000 ms. Relaxation data were acquired from 12,000 echoes. Using MultiExp Inv Analysis software, three primary relaxation components, T_21_, T_22_, and T_23_, were resolved from the curves, and their respective area fractions were quantified as PT_21_, PT_22_, and PT_23_.

MRI analysis of egg white cylindrical gels was performed using the standard spin-echo (SE) sequence, and T1-weighted images were obtained using this NMR instrument. The parameters were set as follows for the measurements: TR = 500 ms; TE = 20 ms. The obtained images were processed using MRI imaging software and displayed in pseudo-color.

#### 2.2.3 Scanning electron microscope (SEM) observation

SEM was used to observe the microstructure of thermally induced gel samples, with slight modifications based on existing methods (Jin et al., 2021). Specifically, thermally induced egg white gel samples were cut into 1 cm^3^ cubic pieces. Subsequently, the gel was washed completely in PBS, followed by dehydration using ethanol at different concentration gradients: 50% (twice), 70%, 80% (twice), 90%, and 100%, each for 20 min. Finally, the sample was lyophilized in a vacuum freeze-dryer and sputter-coated with a layer of gold, followed by observation of egg white thermogel microstructure using an S-4800 SEM (Hitachi Ltd., Tokyo, Japan).

#### 2.2.4 Intermolecular forces measurement

Following a previously described method with minor modifications (Zhang et al., 2022), the intermolecular forces of gel samples were measured. Briefly, a total of 2 g of thermal gel samples were dissolved separately in four solvents: 0.60 M NaCl (S1), 0.60 M NaCl +1.50 M urea (S2), 0.60 M NaCl +8.00 M urea (S3), and 0.60 M NaCl +8.00 M urea +0.50 M β-mercaptoethanol (S4). The mixture was homogenized for 2 min at 12,000 rpm at 4 °C until the gel sample was completely dispersed. Then, the mixture was centrifuged at 10,000 ×*g* for 15 min at 4 °C, and the protein concentration in the supernatant was determined using the Bradford protein assay kit. The solubility of thermogels was governed by distinct forces: S1 by electrostatic interactions; S2 by hydrogen bonds; S3 by hydrophobic interactions; and S4 by disulfide bonds.

#### 2.2.5 Determination of Fourier transformed infrared (FT-IR)

Protein secondary structure was determined using an FTIR spectrometer (Nicolet Nexus 470, Thermo Electron, USA) based on existing methods (Ding et al., 2020). A homogeneous mixture was prepared by combining freeze-dried gel (1 mg) and KBr (100 mg). After thorough grinding, the resulting powder was pressed into thin chips. Then, the secondary structure of the protein gel was assessed using FTIR spectroscopy under test conditions consisting of a scanning range from 400 to 4000 cm^−1^, a resolution of 4 cm^−1^, and 64 scans with an ambient temperature of 25 °C. Changes in the secondary structure composition of EWP gels were analyzed using Peak Fit v4.12 software (SeaSolve, Framingham, USA).

#### 2.2.6 Fluorescence spectroscopy analysis

The fluorescence spectrophotometer (RF-5301, Shimadzu, Tokyo, Japan) was used to characterize changes in the tertiary structure of egg white gels by detecting intrinsic fluorescence intensity (Zang et al., 2025b). The fluorescence intensity was measured by diluting the egg white gel with phosphate buffer (0.1 mol/L, pH 7.0) to a protein concentration of 0.2 mg/mL. The specific parameters were set as follows: excitation wavelength 280 nm, emission wavelength range of 300–450 nm, and slit width 5 nm.

### 2.3 Quantitative analysis of N-glycans in the N-glycoproteome

#### 2.3.1 Protein extraction and digestion

Egg white proteins were extracted following a modified protocol based on Cavallero et al. (2020). The homogenized egg white was diluted 1:1 with Milli-Q water. To precipitate the proteins, an aliquot of the diluted solution was mixed with an equal volume of 50% (*v*/v) trichloroacetic acid and incubated at 25 °C for 2 h. The protein precipitate was collected via centrifugation at 12,000 rpm for 10 min at 4 °C, then cyclically washed and resuspended in Milli-Q water until the supernatant reached a neutral pH. Protein concentrations were then determined using a BCA Protein Assay Kit.

Subsequently, the extracted proteins were dissolved in 8 M urea and diluted with 50 mM NH_4_HCO_3_ to a final urea concentration below 1 M. Disulfide bonds were reduced by incubating the solution with 5 mM dithiothreitol (DTT) at 55 °C for 45 min. After cooling to room temperature, 15 mM iodoacetamide was added, and the protein sample was incubated in the dark for 30 min to alkylate thiol groups. The alkylation reaction was quenched with 5 mM DTT for 15 min. For digestion, trypsin was added at an enzyme-to-protein mass ratio of 1:50 for 18 h, followed by a secondary digestion at a ratio of 1:100 for 4 h. Finally, the digested peptides were desalted using a homemade C18 column, vacuum concentrated, and lyophilized.

#### 2.3.2 Enrichment of intact N-glycopeptides

The Hydrophilic Interaction Liquid Chromatography (HILIC) TopTip™ was activated four times with 150 μL of 0.1% formic acid (FA) and then equilibrated with 150 μL of loading buffer (80% acetonitrile [ACN]/2% trifluoroacetic acid [TFA]). Subsequently, the desalted peptide sample was resuspended in loading buffer and loaded onto the equilibrated HILIC TopTip. Finally, the HILIC TopTip was washed eight times with loading buffer, and the captured glycopeptides were eluted stepwise with 70%, 60%, and 50% ACN (each containing 1% TFA). The samples were then vacuum-dried for LC-MS/MS analysis.

#### 2.3.3 LC-MS/MS analysis

We dissolved the dried intact glycopeptides in 0.1% FA for analysis on an Ultimate 3000 system, which was coupled to an Orbitrap Eclipse mass spectrometer (Thermo Fisher Scientific, San Jose, CA, USA). A binary mobile phase system composed of deionized water containing 0.1% formic acid (A) and 20% deionized water and 0.1% formic acid and 80% acetonitrile (B) was used for all analyses. The flow rate was maintained at 600 nL/min. For intact N-glycopeptide separation, a 120 min gradient was performed with the following sequence: ramp from 4% to 10% B at 4 min, then to 22% B at 84 min, then to 40% B at 104 min, then to 95% B at 110 min, hold at 95% B until 115 min, then drop to 4% B at 120 min. Data-dependent acquisition mode was adopted for Orbitrap Eclipse mass spectrometer detection. Setting parameters as follows: resolution, 60 k; scan range (*m*/*z*), 350–1500; AGC target, 4e^5^; and maximum injection time (IT), 50 ms. The 20 most intense peptide ions were selected for subsequent high-energy collisional dissociation MS/MS scans. The MS/MS settings were as follows: resolution 15 k, an AGC target of 5e^4^, maximum IT 22 ms, and normalized collision energy 30%.

#### 2.3.4 Database searching and data processing

Intact N-glycopeptides were identified by searching the raw MS/MS spectra against the UniProt *Gallus gallus* proteome database using Byonic software (version 3.5.0, Protein Metrics). Search parameters were set as follows: precursor mass tolerance, 20 ppm; MS/MS tolerance, 20 ppm; maximum missed cleavages, 3; with cysteine carbamidomethylation and methionine oxidation. The resulting database was filtered to a 1% false discovery rate (FDR) using 2D-FDR scores, and glycopeptide identifications with Byonic scores below 100 were discarded. The resulting N-glycan compositions were mapped against database entries in the GlyConnect repository (https://glyconnect.expasy.org), where the most accurate N-glycan structural frameworks are available. The N-glycopeptides present in at least one biological replicate were considered for subsequent analysis.

### 2.4 Statistical analysis

To assess statistical significance, one-way ANOVA and Duncan's multiple range tests were executed in IBM SPSS 26.0. The significance level was set at *p* < 0.05. The experimental data were presented as the mean value ± standard deviation. For the identification of significant site-specific N-glycans, a combination of Student's *t*-tests (*p* < 0.05) and a fold-change cutoff (|FC| > 1.5) was applied. Data visualization was performed using R language, OriginPro 2024, and GraphPad Prism 8.00.

## 3 Results

### 3.1 Egg white internal quality and thermogel textural characteristics

We analyzed the albumen rate, albumen height, Haugh units (HU), and texture properties to evaluate the effect of HF on egg white quality during the late laying period, as shown in Fig. 1a-g. Compared with the control group, HF significantly improved egg white internal quality during the late laying period, as evidenced by increased HU and albumen height (*p* < 0.05, Fig. 1b and c), while there was no statistically significant difference in albumen rate between the two groups (*p* > 0.05, Fig. 1a). Meanwhile, as shown in Fig. 1d-g, HF significantly improved egg white thermogel hardness, chewiness, and gumminess in late-laying hens (*p* < 0.01), but had no significant effect on springiness (*p* > 0.05).

**Fig. 1:**
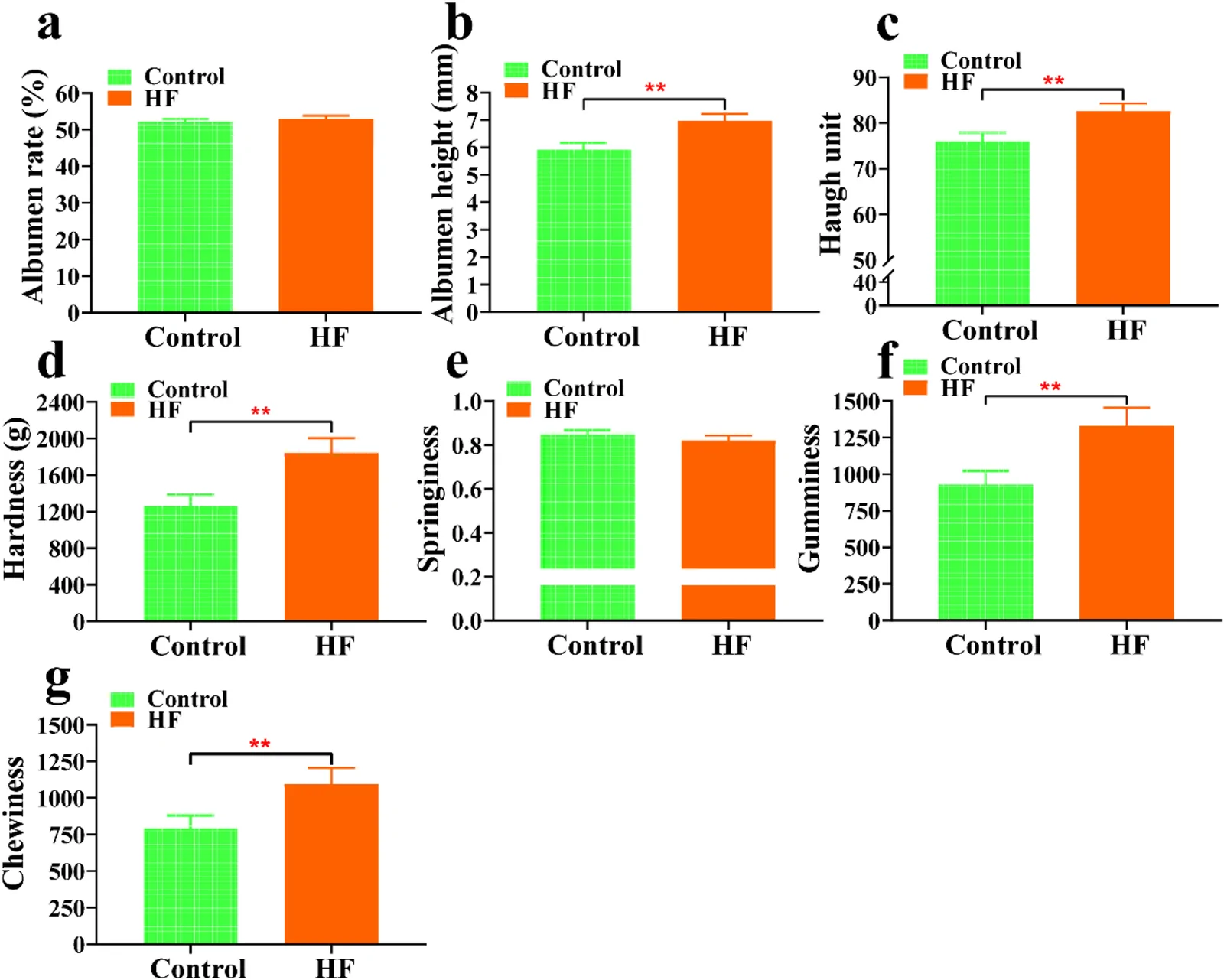
The effects of HF on the gel characteristics of egg white, including albumen rate (a), albumen height (b), and haugh unit (c), and thermal-induced gel texture characteristics including hardness (d), springiness (e), gumminess (f), and chewiness (g). Asterisks indicate significant differences between groups (*P* < 0.05); Double asterisk indicates extremely significant difference (*P* < 0.01).

### 3.2 WHC and moisture distribution in egg white thermal gels

This study found that, compared to the control group, a remarkable improvement in the WHC of egg white gels was observed in the HF treatments (Fig. 2a). Typically, a higher gel strength is associated with better water retention, indicating that adding HF enhanced moisture retention within egg white gels, aligning with the gel strength findings shown in Fig. 1d-g. Concurrently, we further utilized LF-NMR to evaluate the state and distribution of water molecules in egg white gels. As shown in Fig. 2b, the transverse relaxation curves of egg white gel presented three T_2_ distribution peaks, corresponding to the distribution of bound water (0.1–1.0 ms), immobilized water (10.0–100.0 ms), and free water (>100.0 ms), respectively. The peak area of each T_2_ distribution peak indicated the relative content of the water state. The dominant T₂₂ peak confirmed that immobilized water predominates in egg white thermogel during the late laying period. Compared with the control group, the distribution peak of the T₂₂ curve in egg white gels with HF shifted toward lower relaxation times, reflecting reduced water mobility (Fig. 2b). Meanwhile, as presented in Table S1, HF supplementation significantly elevated the PT_22_ proportion and reduced the PT_23_ proportion of egg white gels (*p* < 0.05), suggesting an enhanced conversion of free water to immobilized water during gelation.

**Fig. 2:**
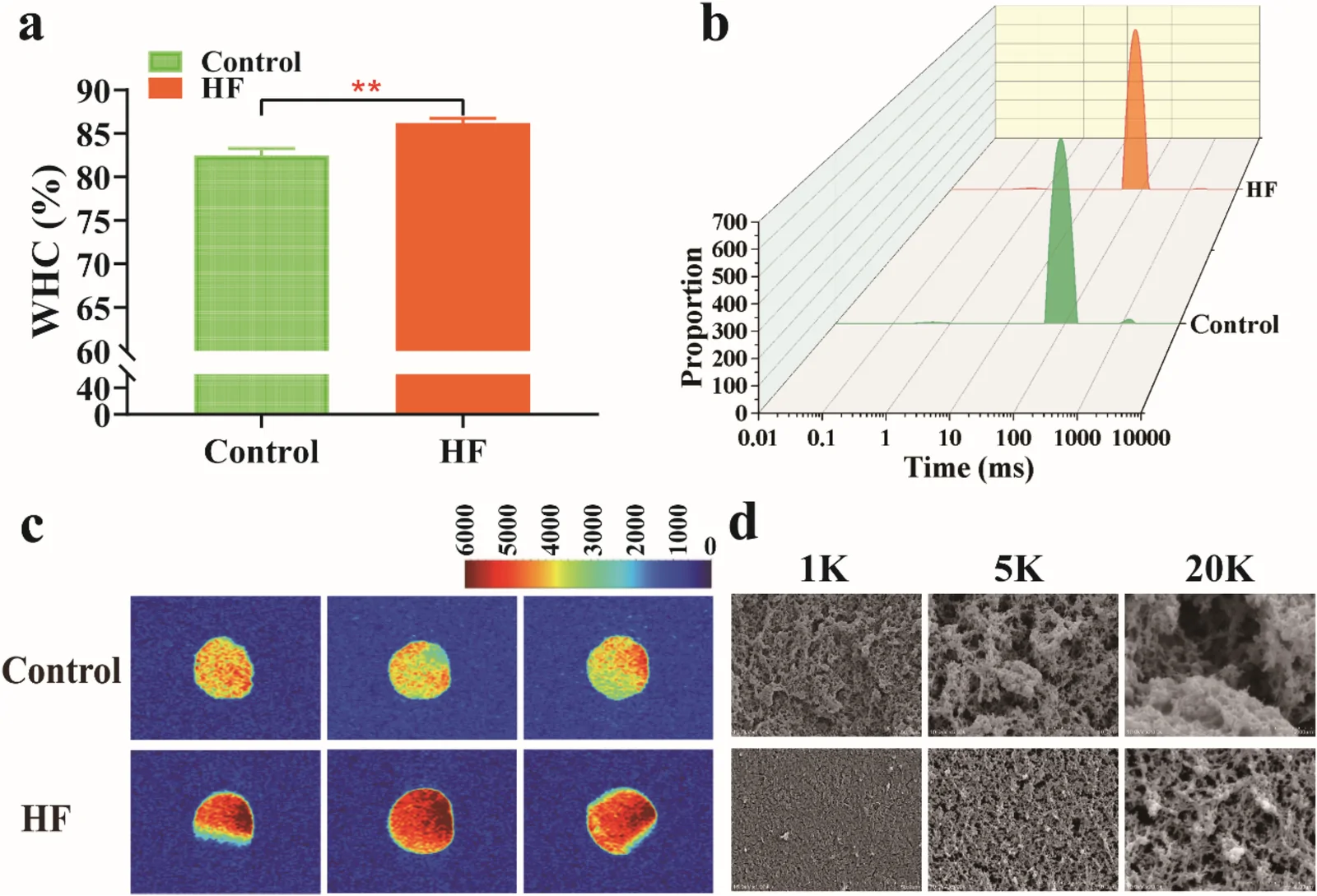
The effects of HF on the WHC (a), transverse relaxation curves (b), T1-weighted images (c), and microstructure (d) of heat-induced egg white gels.

To further elucidate changes in the spatial distribution and mobility of water within heat-induced egg white gels, we employed a non-invasive, non-destructive, high-resolution MRI technique to observe egg white thermogels during the late laying period. In T1-weighted images, the density of protons with high relaxation time was lower, whereas the density of protons with low relaxation time was brighter, and the pseudo-color images were redder. This study found that the addition of HF produced a notably stronger red intensity on the T1-weighted image of egg white gels (Fig. 2c), indicating enhanced water immobilization and water retention, as well as reduced moisture migration in egg white thermogels. This result aligns well with the findings from LF-NMR relaxation time and WHC analyses.

### 3.3 Changes in egg white thermogel microstructure

The 3D microstructure and spatial conformation of thermally induced egg white gel can be visualized employing SEM. As can be observed, the fractured cross-section of egg white thermogel from late-laying hens showed a loose, more porous network structure characterized by large protein aggregates and poorly distributed macropores (Fig. 2d). At the same time, the section surface of egg white gel samples with HF addition exhibited a more compact and ordered gel network structure, with uniformly distributed small pores and compactly aggregated small protein particles, further substantiating the earlier evidence on the enhancement of gel network density.

### 3.4 Quantitative analysis of site-specific N-glycan features in the N-glycoproteome

#### 3.4.1 Deep characterization of intact N-glycopeptides from egg white protein

This study systematically characterized site-specific N-glycan structural features for EWPs to clarify the site distribution and N-glycan heterogeneity of N-glycosylation modifications in egg white during the late laying period. We employed a label-free glycoproteomic strategy and successfully identified 767 unique intact N-glycopeptides from 4856 high-quality MS/MS spectra. These corresponded to 76 distinct N-glycan compositions and 87 unique N-glycosites distributed across 46 N-glycoproteins in egg white (Fig. 3a). High-quality MS/MS spectra for intact N-glycopeptides bearing extended N-glycan chains are shown in Fig. 3b, c and Fig. S1, demonstrating successful identification of distinct site-specific N-glycan features. Moreover, of the glycosites identified in this study, approximately 50.6% were annotated in the UniProt database (Fig. 4a). Besides 20 previously verified glycosites, we provided experimental evidence for 24 UniProt-predicted glycosites (21 sequence analyses and 3 by similarity) and 43 non-Uniprot-recorded glycosites (Fig. 4a). Furthermore, sequence motif analysis of our identified N-glycosites predominantly featured N-X-S (about 36%) and N-X-T (about 56%) motifs, whereas only about 6% of the glycosites carried the N-X-C motif, and HF had no effect on these sites (Fig. 4b and c). This suggests that N-glycosylation in the N-X-T sequence is more prevalent in egg white glycoproteins. To further comprehensively characterize the N-glycan features of EWPs in late-laying hens, we subjected the top 5 N-glycans with the highest relative abundance for comparative analysis, finding that the undecorated Hex_3_HexNAc_6_ (No.1) exhibited the highest abundance, followed by Hex_4_HexNAc_6_, Hex_3_HexNAc_5_, Hex_3_HexNAc_7_, and Hex_3_HexNAc_8_. Meanwhile, HF significantly increased the relative abundance of undecorated Hex_3_HexNAc_7_, Hex_4_HexNAc_7_, Hex_5_HexNAc_4_, and Hex_4_HexNAc_5_, whereas it significantly decreased the relative abundance of undecorated Hex_3_HexNAc_6_, Hex_4_HexNAc_6_, Hex_3_HexNAc_5_, and Hex_3_HexNAc_4_ (Fig. 4d).

**Fig. 3:**
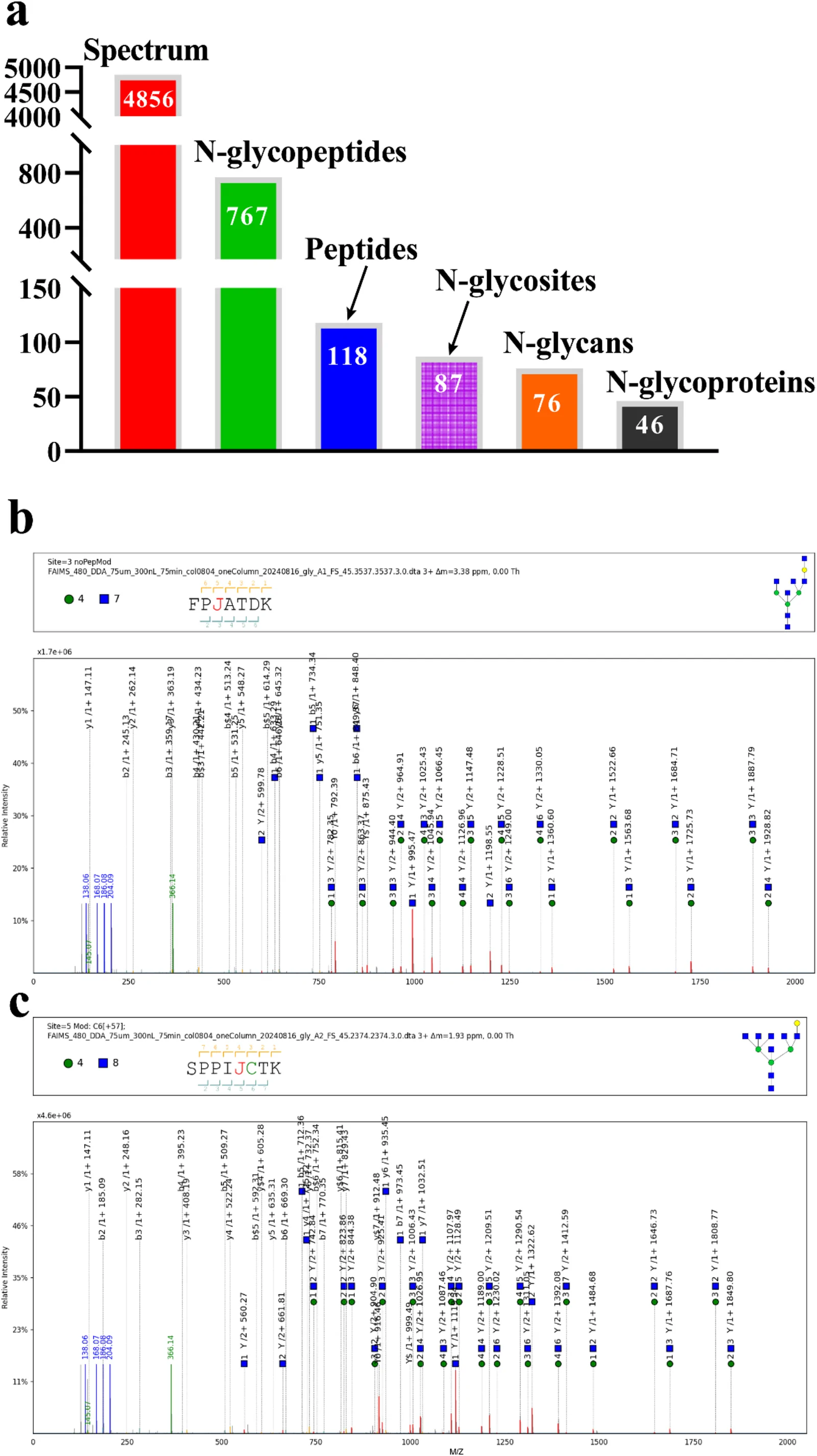
Site-specific glycoproteomics analysis of EWP. (a) the number of intact N-glycopeptides, N-glycosites, N-glycans, and N-glycoproteins in EWP. (b-c) Examples of the MS/MS spectra of the intact N-glycopeptides: Ovomucoid (P01005) with “Hex_4_HexNAc_7_” (b) and Mucin-5B (Q98UI9) with “Hex_4_HexNAc_8_” (c).

**Fig. 4:**
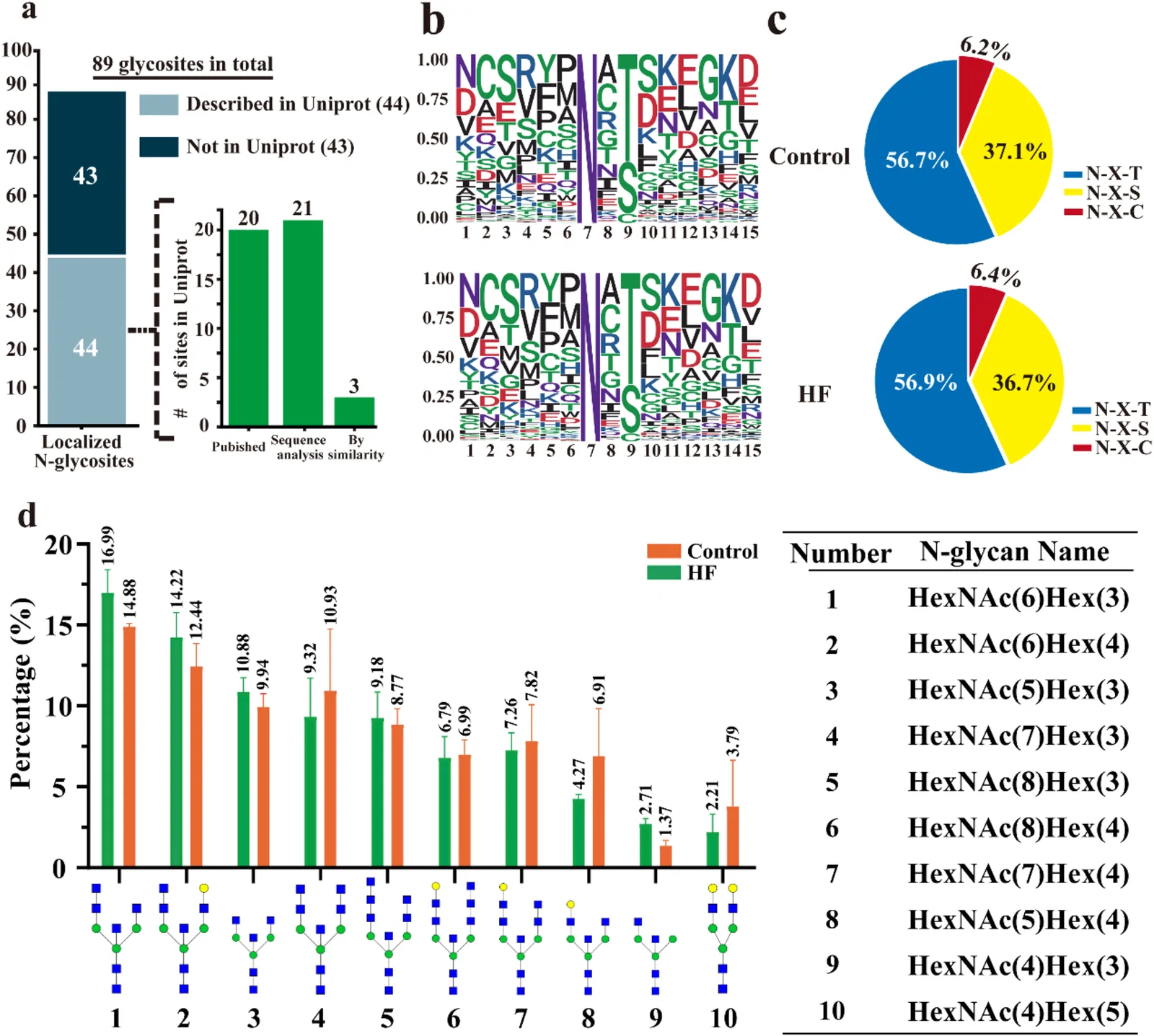
The status of quantified glycosites recorded in the UniProt database (a). Recognition of the sequence motif of N-glycosylation (N-X-S/T/C, where N is asparagine, X is any amino acid except proline, S is serine, T is threonine, and C is cysteine) and their relative percentage in the unique glycosites identified between control and HF groups (b and c). Relative abundance of the top ten abundant N-glycans identified from egg white glycoproteins between control and HF groups. Green circles, yellow circles, blue squares, red triangles, and purple diamonds represent mannose, galactose, HexNAc, fucose, and Neu5Ac (d). (For interpretation of the references to color in this figure legend, the reader is referred to the web version of this article.)

#### 3.4.2 Overall structural characterization of site-specific N-glycan features in egg white glycoproteins

To further characterize the overall structural characterization for site-specific N-glycan features in EWPs in late-laying hens, we classified identified N-glycans into six categories based on distinct glycosyl modifications, as undecorated (neither sialylated nor fucosylated), high-mannose, paucimannose, sialylated-only, fucosylated-only, and sialofucosylated (both sialylated and fucosylated) modifications. Among these, undecorated N-glycans dominated, accounting for approximately 60%, followed by paucimannose and high-mannose N-glycans, with sialofucosylated N-glycans being the lowest proportion at merely about 0.6% (Fig. 5a). Meanwhile, HF increased the proportion of paucimannose and sialylated-only N-glycans, while decreasing the proportion of undecorated and high-mannose N-glycans in egg white in late-laying hens (Fig. 5a).

**Fig. 5:**
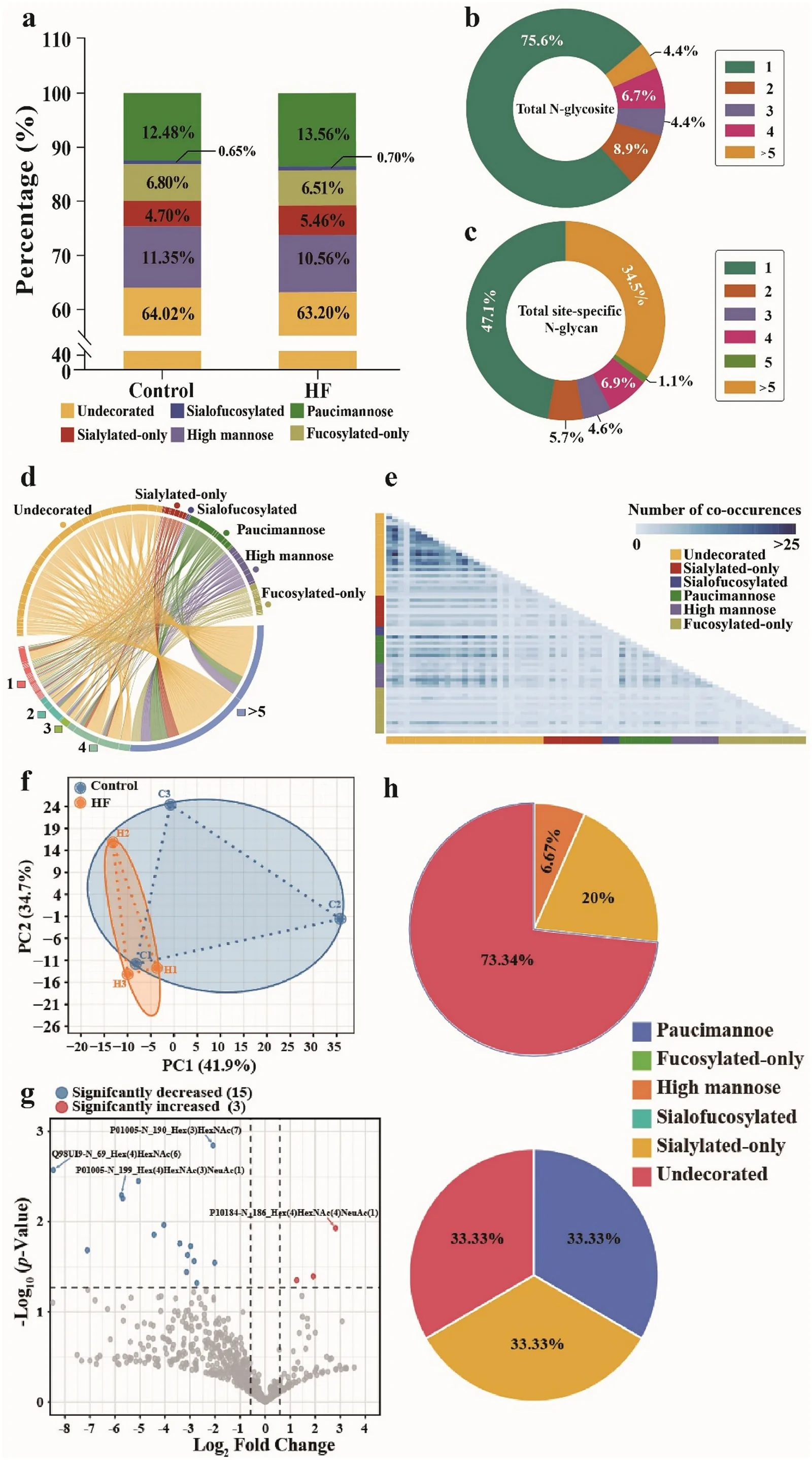
Comprehensive characterization of site-specific N-glycans in egg white proteins. Relative abundance of N-glycans types identified among control and HF groups (a); The number and percentage of N-glycosites per glycoprotein identified (b); Distribution of the number and percentage of different glycans seen at a given glycosite (c); A glycoprotein-glycan network maps which glycans (top) modify which proteins (bottom) (d); A glycan co-occurrence heat map represents the number of times glycan pairs appeared together at the same glycosite, indicating which glycans contribute most to microheterogeneity that had more than one glycan modifying them (e). PCA score plot of egg white proteins site-specific N-glycosylation in control and HF groups (f); Volcano diagram for differentially changed site-specific N-glycans of egg white glycoproteins in control and HF groups. Red circles: Significantly increased site-specific N-glycans with fold change >2 (*p*-value <0.05). Blue circles: Significantly decreased site-specific N-glycans with fold change <0.5 (*p*-value <0.05) (g); Type distribution of significantly increased (Bottom) and decreased (Top) site-specific N-glycans (h). (For interpretation of the references to color in this figure legend, the reader is referred to the web version of this article.)

#### 3.4.3 Analyzing egg white N-glycoproteomic heterogeneity

We uniquely characterized the heterogeneity of N-glycosylation modifications in EWPs with intact N-glycopeptide analysis, contributing to a deeper comprehension of the structural and functional characteristics in egg white glycoproteins. Macroheterogeneity refers to the phenomenon that a single protein contains more than one N-glycosite. As depicted in Fig. 5b, the majority of identified egg white glycoproteins (75.6%) possessed a single N-glycosite, whereas a substantial proportion (24.4%) harbored multiple sites. A prominent example is α-ovomucin (mucin-5B), which was found to carry as many as 18 distinct N-glycosites (Fig. 6b). Furthermore, microheterogeneity refers to the presence of multiple N-glycans attached to a single N-glycosite. According to Fig. 5c, 52.9% of N-glycosites in egg white glycoproteins from late-laying hens contained over one N-glycan, while 34.5% harbored over five N-glycans. Table S2 summarizes the glycosylation sites of EWPs that harbor over five site-specific N-glycan features, with a large number of site-specific N-glycans identified on egg white glycoproteins such as ovomucoid, ovotransferrin, and mucin-5B. Of these, compared to the control group, HF markedly reduced the number of site-specific N-glycans at the N190, N199, and N77 sites on ovomucoid. Specifically, the number of N-glycans at these three glycosites decreased from 32 to 26 N-glycans, 34 to 31 N-glycans, and 22 to 13 N-glycans, respectively.

**Fig. 6:**
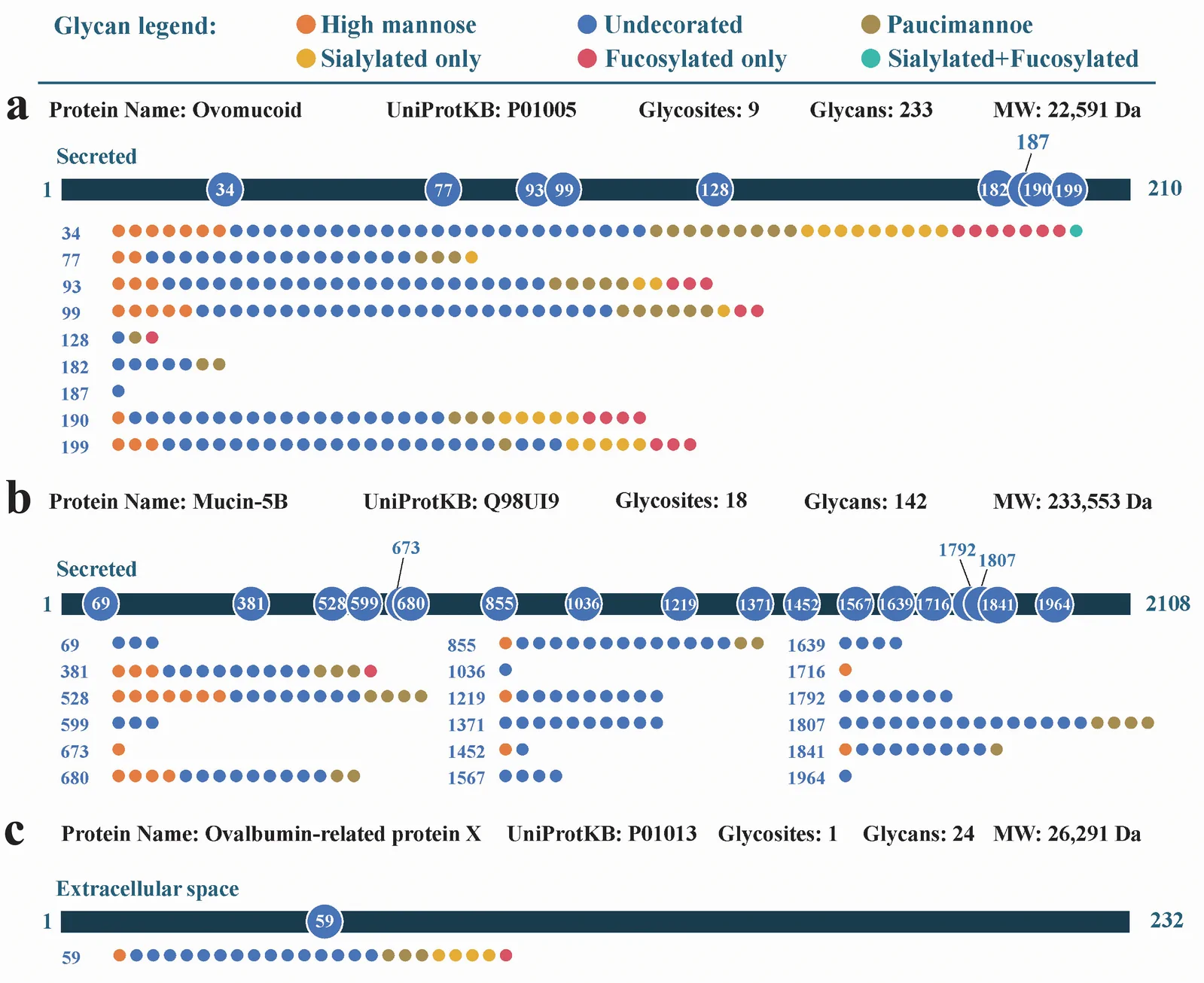
Glycan microheterogeneity can manifest in several different forms. The site-specific N-glycan distribution of Ovomucoid (a), Mucin-5B (b), and Ovalbumin-related protein X (c) in egg white proteins from laying hens.

The network diagram of N-glycan types and N-glycosites (Fig. 5d) and the heatmap displaying the frequency of N-glycan pairs co-occurring at the same site on glycoproteins (Fig. 5e) were used to further investigate site-specific N-glycan microheterogeneity in egg white glycoproteins. The network analysis demonstrated that undecorated N-glycans were ubiquitously distributed across egg white glycoproteins. Notably, fucosylated-only and sialylated-only N-glycans were more frequently present on egg white glycoproteins carrying multiple N-glycosites (Fig. 5d). The heatmap further indicated that undecorated N-glycans co-occurred together frequently at the same site, and they also co-occurred with high-mannose, paucimannose, and sialylated-only N-glycans, underscoring the complexity of site-specific microheterogeneity in egg white glycoproteins (Fig. 5e). Moreover, our analysis of individual glycoproteins revealed distinct patterns of microheterogeneity. On one hand, ovomucoid harbored numerous N-glycosites but relatively little N-glycan heterogeneity overall (Fig. 6a), whereas ovalbumin-related protein X possessed only a single N-glycosite with multiple N-glycans modifying it (Fig. 6c). For instance, high-mannose, paucimannose, undecorated, sialylated-only, and fucosylated-only N-glycans were all observed on N34, N93, N99, N190, and N199 of ovomucoid (Table S2). On the other hand, some N-glycosites on mucin-5B displayed little heterogeneity, whereas other sites harbored 10–19 distinct glycan modifying them (Fig. 6b). For example, mucin-5B only carried undecorated N-glycan on N69, N599, N1036, N1371, N1567, N1639, N1792 and N1964, while high-mannose, paucimannose, and undecorated N-glycan were carried at N528, N680, N855 and N1841 (Table S2).

#### 3.4.4 Deep mining of changes in N-glycan features for egg white glycoprotein between control and HF groups

To further explore how adding HF influences the N-glycan features in EWPs during the late laying period, we quantified intact N-glycopeptides employing an N-glycoproteomic approach. Principal component analysis (PCA) showed clear separation between the HF and control groups along the PCA1 axis, suggesting that HF supplementation markedly altered the N-glycan features in egg white glycoproteins (Fig. 5f). It was observed that HF significantly decreased 15 site-specific N-glycan structures, which corresponded to 5 N-glycosylation sites across 4 egg white glycoproteins, among which Hex_4_HexNAc_6_ at the N69 site of Q98UI9 showed the most pronounced effect. Conversely, HF notably elevated 3 site-specific N-glycan structures corresponding to 3 N-glycosylation sites across 3 egg white glycoproteins, among which Hex_4_HexNAc_4_NeuAc_1_ at the N186 site of P10184 exhibited the most pronounced increase (Fig. 5g). Among the site-specific N-glycans significantly increased in the HF group, 73.34% were undecorated and 20% were sialylated only, whereas, among site-specific N-glycans significantly decreased, 33.3% were undecorated and 33.3% were sialylated only (Fig. 5h). Among these, significantly altered site-specific N-glycans were primarily observed at the mucin-5B, ovomucoid, and ovotransferrin. This research discovered a significant decrease in the abundance of Hex_4_HexNAc_6_ and Hex_3_HexNAc_6_ attached to the N69 site of mucin-5B in the HF group compared to the control group (Table 1). Similarly, HF markedly decreased the abundance of high-complexity N-glycans (highly branched and heavily sialylated) on ovomucoid N199 and N190, such as Hex_4_HexNAc_3_NeuAc_1_, Hex_3_HexNAc_7_, and Hex_5_HexNAc_6_ (*p* < 0.05, Table 1). Conversely, HF significantly increased the abundance of Hex_9_GlcNAc_2_, Hex_3_HexNAc_7_, and Hex_4_HexNAc_4_ N-glycans on ovotransferrin (OVT) N492 (Table 1).

**Table 1:** Significantly Increased and Decreased Site-Specific N-Glycans in the Control Compared with HF.

Tag^a^	Protein	Plausible structure^b^	Type	Change (Genistein /Control)	Fold change (B/A)	*P*-value (B/A)
Q98UI9-N_69	Mucin-5B		Undecorated	Decreased	1.07E-18	2.51E-03
I0IUP4-N_150	DNA helicase MCM9		Fucosylated-only	Decreased	3.42 E-18	7.89E-02
Q98UI9-N_69	Mucin-5B		Undecorated	Decreased	7.26E-03	2.07E-02
Q98UI9-N_1371	Mucin-5B		Undecorated	Decreased	7.37E-03	5.65E-02
Q98UI9-N_1371	Mucin-5B		Undecorated	Decreased	9.01E-03	7.42E-02
Q98UI9-N_1371	Mucin-5B		Undecorated	Decreased	1.27E-02	9.29E-02
P01005-N_199	Ovomucoid		Sialylated-only	Decreased	1.88E-02	5.08E-03
P01005-N_190	Ovomucoid		Sialylated-only	Decreased	1.94E-02	5.50E-03
Q98UI9-N_1371	Mucin-5B		Undecorated	Decreased	1.94E-02	8.79E-02
P01005-N_190	**Ovomucoid**		Fucosylated-only	Decreased	2.44E-02	6.97E-02
P01012-N_293	**Ovalbumin**		Undecorated	Decreased	2.99E-02	3.53E-03
P01005-N_199	**Ovomucoid**		Undecorated	Decreased	4.59E-02	1.39E-02
P01005-N_199	**Ovomucoid**		Sialylated-only	Decreased	5.23E-02	6.89E-02
P01005-N_190	**Ovomucoid**		Undecorated	Decreased	5.54E-02	9.79E-02
P01005-N_190	**Ovomucoid**		Fucosylated-only	Decreased	5.59E-02	6.69E-02
P01005-N_190	**Ovomucoid**		Undecorated	Decreased	6.09E-02	1.09E-02
P01005-N_199	**Ovomucoid**		Undecorated	Decreased	8.16E-02	9.87E-02
P01005-N_190	**Ovomucoid**		Sialylated-only	Decreased	8.41E-02	7.11E-02
P01005-N_190	**Ovomucoid**		Undecorated	Decreased	9.03E-02	9.96E-02
P01005-N_190	**Ovomucoid**		Undecorated	Decreased	9.04E-02	1.75E-02
P01005-N_190	**Ovomucoid**		Undecorated	Decreased	1.12E-01	9.86E-02
P01005-N_199	**Ovomucoid**		Undecorated	Decreased	1.13E-01	3.61E-02
P01005-N_199	**Ovomucoid**		Undecorated	Decreased	1.16E-01	2.34E-02
Q98UI9-N_528	Mucin-5B		Undecorated	Decreased	1.19E-01	9.70E-02
P01005-N_199	**Ovomucoid**		Sialylated-only	Decreased	1.26E-01	1.86E-02
P01005-N_199	**Ovomucoid**		Undecorated	Decreased	1.30E-01	7.99E-02
P01005-N_190	**Ovomucoid**		Undecorated	Decreased	1.38E-01	6.03E-02
P01005-N_199	**Ovomucoid**		Undecorated	Decreased	1.39E-01	2.74E-02
P01014-N_95	**Ovalbumin-related protein Y**		High mannose	Decreased	1.51E-01	4.77E-02
P01005-N_199	**Ovomucoid**		Undecorated	Decreased	1.61E-01	6.99E-02
P01013-N_59	**Ovalbumin-related protein X**		Sialylated-only	Decreased	1.70E-01	8.20E-02
P20740-N	**Ovostatin**		Paucimannose	Decreased	1.89E-01	9.03E-02
P01005-N_199	**Ovomucoid**		Undecorated	Decreased	1.95E-01	5.85E-02
P01005-N_190	**Ovomucoid**		Undecorated	Decreased	2.36E-01	1.42E-03
P01005-N_182	**Ovomucoid**		Undecorated	Decreased	2.39E-01	6.95E-02
Q98UI9-N_528	Mucin-5B		Undecorated	Decreased	2.40E-01	7.33E-02
P01005-N_199	**Ovomucoid**		Undecorated	Decreased	2.47E-01	2.85E-02
P02789-N_492	Ovotransferrin		High mannose	Increased	4.63E+16	7.42E-02
P10184-N_186	Ovoinhibitor		Sialylated-only	Increased	7.02E+00	1.17E-02
P01005-N_199	**Ovomucoid**		Paucimannose	Increased	3.79E+00	4.03E-02
P02789-N_492	Ovotransferrin		Undecorated	Increased	2.77E+00	6.64E-02
P02789-N_492	Ovotransferrin		Undecorated	Increased	2.40E+00	4.46E-02

a Tags consist of protein UniProt ID and N-glycosite.

b Green circles, yellow circles, blue squares, red triangles, yellow square, and purple diamonds represent mannose, galactose, GlcNAc, fucose, GalNAc, and Neu5Ac.

### 3.5 Alterations of secondary, tertiary structures and intermolecular forces in egg white thermal gels

Fourier transform infrared spectroscopy was an effective approach for investigating changes in the structures of egg white protein gels. For FT-IR determination of protein secondary structure, the amide I region (1600–1700 cm^−1^) is most informative. Within this band, ordered structures are assigned as follows: β-sheet (1610–1639 cm^−1^), α-helix (1650–1659 cm^−1^), and β-turn (1660–1700 cm^−1^), while random coil (1640–1649 cm^−1^) represents the disordered fraction. Gaussian fitting and second-derivative analysis (Fig. 7a and b) revealed that the secondary structures of egg white gel samples in two groups were predominantly β-turn and β-sheet, whereas the proportions of α-helix and random coils were relatively low. Notably, adding HF significantly increased the content of α-helix and β-sheet in egg white gels, whereas the random coil content was markedly reduced (*p* < 0.05, Fig. 7c). This suggests that HF promoted the secondary structure transition from random coils to α-helices and β-sheets in egg white gels in late-laying hens.

**Fig. 7:**
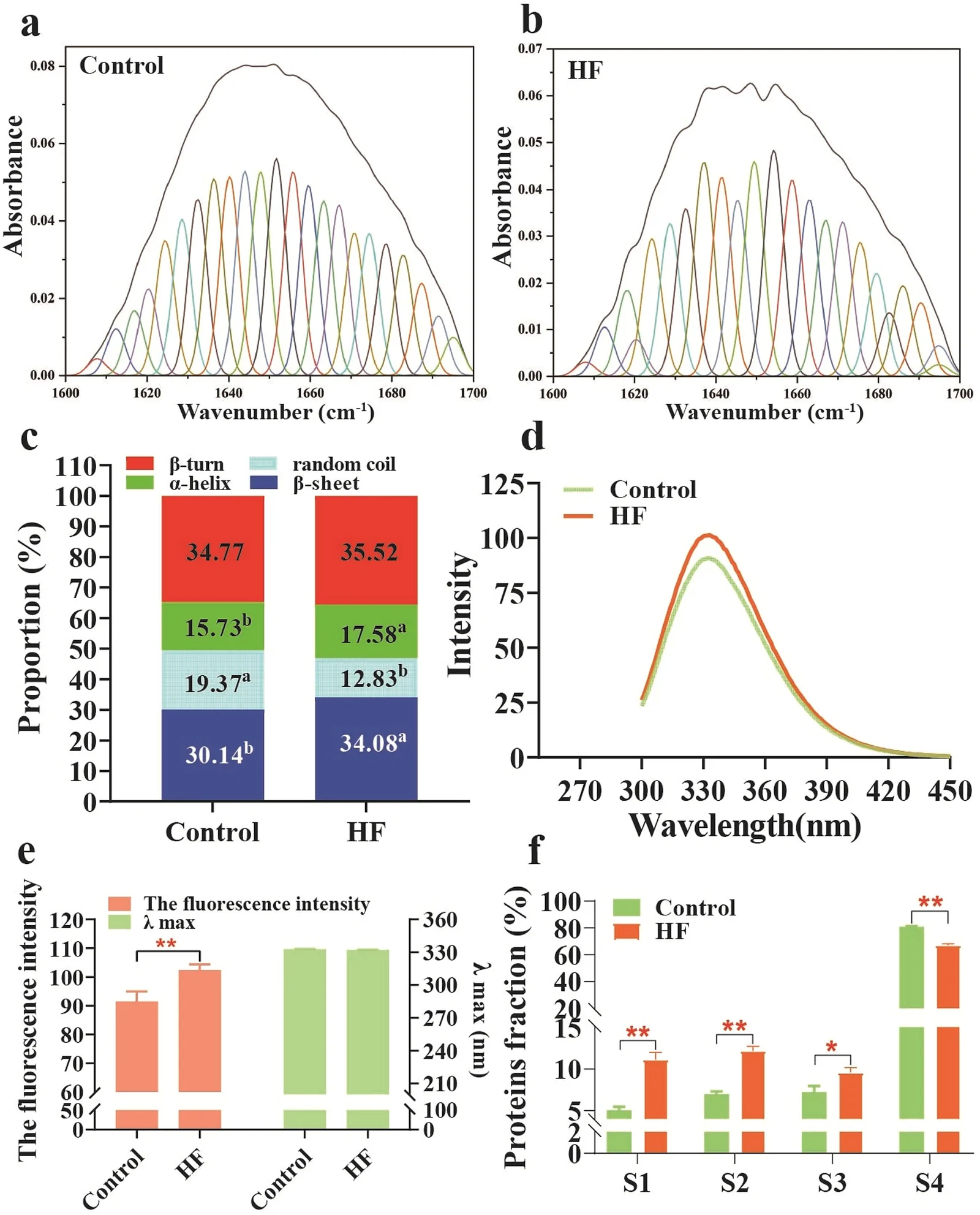
The effects of HF on the peak diagrams of Gaussian fitting curves in the amide I region (a and b), secondary structure (c), intrinsic fluorescence intensity (d), and fluorescent maximum wavelength (λ max) of egg white protein (e). The effects of HF on the intermolecular forces of egg white heat-induced gel (f). Asterisks indicate significant differences between groups (*P* < 0.05); Double asterisk indicates extremely significant difference (*P* < 0.01).

In addition, changes in intermolecular interactions involving hydrogen bonds, ionic bonds, disulfide bonds, and hydrophobic interactions were commonly regarded as primary reasons for alterations in thermogel characteristics of proteins. Fig. 7f reveals that HF significantly enhanced hydrophobic interactions, hydrogen bonds, and ionic bonds within thermal gels (*p* < 0.05), whilst markedly reducing disulfide bond formation (*p* < 0.05).

We further probed the tertiary structure changes of egg white protein gels using intrinsic fluorescence spectroscopy, which primarily arises from electronic transitions in tryptophan (Trp), tyrosine (Tyr), and phenylalanine (Phe) residues. Compared with the control group, intrinsic fluorescence spectroscopy revealed that HF remarkably increased the intrinsic fluorescence intensity of egg white gels without altering the maximum fluorescence wavelength (λ max) (Fig. 7d and e).

## 4 Discussion

Egg white is widely utilized in the food industry due to its unique gelling, foaming, and emulsifying properties, particularly its excellent heat-induced gelation ability, which critically determines the texture, mouthfeel, and WHC of processed egg products (Alavi et al., 2020). However, the gelling properties of egg whites rapidly decline with extended laying cycles, severely limiting their processing and utilization value (Chang et al., 2024; Li et al., 2023). Recently, numerous studies have shown that flavonoids can improve the internal quality and gelling properties of egg white by regulating the composition, abundance, and glycosylation levels of EWPs (Li, Chen, et al., 2025; Liu et al., 2021; Zhong et al., 2022; Zhou et al., 2025). Meanwhile, our previous research has shown that HF improved egg quality in late-laying hens by modulating the morphological structure and protein synthesis capacity of oviducts (Wang et al., 2025). Notably, more than 80% of EWPs are glycosylated, and the main EWPs (such as ovalbumin, ovomucin, and ovotransferrin) exist as glycoproteins. Among these, N-glycosylation is the most prevalent post-translational modification of proteins, occurring in over 70% of glycoproteins (Guan et al., 2023; Yu et al., 2025). Studies have shown that N-glycosylation can alter egg white protein structure, stability, and interactions, thereby affecting its functional properties, including thinning, antimicrobial, and gelling characteristics (Cavallero et al., 2020; Hu et al., 2023). Furthermore, variations in the location and length of glycan chains stemming from differences in N-glycosites and the attached N-glycan chain can alter the three-dimensional structure and molecular interactions of EWPs, thereby affecting the functional properties of EWPs during food processing (Qi et al., 2024; Yu et al., 2025).

Therefore, this study employed N-glycoproteomics to investigate the regulatory effects of HF on the N-glycosites and N-glycan structures of egg white protein, and to elucidate the intrinsic relationship between these effects and the thermal gelation characteristics of EWPs. The results of the present study demonstrated that HF significantly improved egg white internal qualities (HU and albumen height) as well as its thermal gel strength, network structure, and WHC. HU is a critical indicator for evaluating egg white freshness, colloidal stability, and internal quality. A higher HU value indicates a higher viscosity, greater freshness, and better egg white internal quality (Chang et al., 2024; Shuo et al., 2025). With increasing age of the laying hens, egg white undergoes excessive thinning accompanied by reduced thick albumen height, which consequently leads to a decline in HU (Li et al., 2023). Thus, the significant increase in HU in the HF group demonstrated that HF effectively alleviated the excessive egg white thinning and internal quality deterioration during the late laying period. Likewise, quercetin, genistein, and serine have been shown to bolster egg white gel properties through increased microfilament protein content or modulation of egg white protein glycosylation (Li, Chen, et al., 2025; Li, Guan, et al., 2025; Zhou et al., 2025). Meanwhile, texture characteristics, particularly hardness, springiness, chewiness, and gumminess, play a vital role in determining the palatability and mouthfeel of egg white gels by mimicking the chewing process (Zang et al., 2025a). Furthermore, evidence indicates that the HU decline correlated directly with ovomucin glycosidic bond cleavage, which induced structural changes in ovomucin and ultimately reduced its viscoelasticity and thermally induced gelling properties (Li et al., 2023; Shan et al., 2020). Given these findings, we concluded that the improvement in the thermal gelation properties and internal quality of egg whites with HF was closely related to altered protein composition and glycosylation modifications.

The WHC reflects the ability of heat-induced egg white gels to retain moisture, which depends largely on pore size and the structural integrity of the gel network (Zang et al., 2025b; Zhang, Zhao, et al., 2024). The high WHC of gels resulted from abundant stable immobilized water, which was effectively held by capillary forces in small uniform pores, thereby increasing WHC (Farjami et al., 2015; Zhang, Zhao, et al., 2024). In addition, studies have shown that a finer network structure arises from the conversion of free water into bound water, resulting in enhanced gel properties and WHC (Li et al., 2023; Zhang et al., 2022). Similarly, Zhang, Mei, et al. (2024) found that adding ethanol extract of propolis effectively improved the WHC and gel strength of heat-induced egg white gels. This research demonstrated that HF notably improved WHC and immobilized water content of egg white thermogels in late-laying hens, suggesting the formation of a compact gel network with uniformly distributed small pores.

Thus, the microstructural variations of egg white gels were further characterized using SEM. It was observed that HF resulted in a more compact and ordered gel network, accompanied by uniformly distributed small pores. Smaller pore sizes in heat-induced gel structures promote better connectivity within gel network structures, thereby facilitating the formation of thermogels with excellent textural properties and WHC (Han et al., 2014; Jin et al., 2021). Notably, gel network density and connectivity depend critically on the equilibrium between protein unfolding and aggregation rate kinetics during heating (Grossi et al., 2016). Decelerating the protein aggregation rate facilitates the formation of soluble aggregates, thereby promoting the development of a denser, more ordered, and highly cross-linked gel network (Li et al., 2023). Together, we concluded that adding HF slowed egg white protein aggregation rates during thermal induction, consequently enhancing structural integrity and decreasing the porosity of thermal gels. As a result, more immobilized water was retained, which ultimately enhanced the gel performance and WHC of egg whites during the late laying period.

Protein N-glycosylation, a key and widespread post-translational modification, plays a pivotal role in regulating protein solubility, aggregation behavior, and functional properties (Qi et al., 2024). Moreover, variations in glycosite numbers, glycan structures, or glycoprotein abundances have been shown to directly affect egg white internal quality, gelling, and rheological performance (Li et al., 2023; Liu et al., 2021; Zhou et al., 2025). Hence, for advanced investigation on how HF influenced the N-glycan features of EWPs, we employed N-glycoproteome to present the overall glycan structure atlas and heterogeneity of EWPs in late-laying hens. We accurately identified 76 distinct N-glycans, 46 N-glycoproteins, and 87 N-glycosites in EWPs. This research usefully supplemented and broadened prior findings, and further enriched the N-glycoproteome information dataset for egg whites. Meanwhile, we have indeed characterized distinct N-glycan pools and provided evidence demonstrating that the glycoproteome exhibited differing degrees of macro- and microheterogeneity at the glycosite, glycoprotein, and co-occurring glycan levels. This indicated that the glycoproteome of egg whites possessed widespread structural diversity and complexity at the N-glycan level, enabling multiple different types of N-glycan chains to be attached simultaneously to a single glycoprotein molecule (Zhang et al., 2025). From the perspective of heat-induced gel formation, this heterogeneity is not merely structural redundancy but rather a key molecular basis for the functional regulation of glycoproteins. Zhang et al. (2026) discovered that removal of N-glycan chains markedly decreased the cross-linking density in ovomucin gel networks, suggesting that the diversity of glycan chains may directly influence the crosslinking efficiency and network density of egg white gels.

Paucimannose is an essential isoform of N-linked glycans and serves as a “truncated version” of the high-mannose type (Loke et al., 2016). High-mannose N-glycans are one of the basic categories of N-linked glycans, characterized by the attachment of 2 to 9 mannose residues to a core pentasaccharide (Man₃GlcNAc₂), resulting in a mannose-enriched glycan structure (Wang et al., 2018). Numerous studies have confirmed that high-mannose N-glycans perform a critical protective role in glycoprotein folding and thermal stability (Hirose et al., 2024; Tams & Welinder, 2001). Evidence has shown that a significant decrease in paucimannose of glycoproteins indicates a disruption in high-mannose processing, thereby impeding protein folding and thermal stability (Zhang et al., 2025). Concurrently, sialic acid, primarily present as *N*-acetylneuraminic acid (Neu5Ac) in the terminal monosaccharide of N-glycans, is known to protect against premature proteolytic degradation and enhance protein thermal stability through spatial steric hindrance effects (Yang et al., 2024). Our study discovered that HF notably promoted high-mannose processing and sialic acid modification in EWPs, thereby enhancing the stability and functional properties of egg white in late-laying hens. Halder et al. (2015) also found that high-mannose glycans could anchor glycans to protein surfaces by forming hydrophobic interactions and hydrogen bonds with surface residues via the terminal GlcNAc residue, thereby reducing the rate of protein thermal aggregation. Higher natural glycosylation modification of ovomucin has been reported to supply more hydrogen bonds during gelation, thus improving gel stability (Zhang, Zhao, et al., 2024). Meanwhile, the glycosylation of myofibrillin can increase hydrophobic interactions between protein molecules, promoting the formation of an ordered gel network (Ma et al., 2023; Xu et al., 2018). Moreover, sialylated N-glycans can modulate the spatial distances between protein molecules through electrostatic interactions, maintaining protein-protein interaction distances within a range conducive to the formation of cross-linked networks, thereby promoting the formation of ordered protein gel networks (Zhang et al., 2026). Combining the results of intermolecular interactions, we concluded that HF can promote the formation of more ionic bonds, hydrophobic interactions, and hydrogen bonds between protein molecules by increasing paucimannose and sialylated N-glycans in EWPs, thereby ameliorating the thermal gelling characteristics of EWPs in late-laying hens.

The glycosylation modifications of main proteins in egg whites, such as mucin-5B, ovomucoid, and ovotransferrin, were tightly associated with the thermal gelation characteristics of EWPs and could modulate egg white gel properties through steric hindrance effects of glycan chains (Liu et al., 2021; Liu, Wang, et al., 2022; Zhang et al., 2019). Mucin 5B possessed numerous N-glycosites, and its glycan structures critically influenced the conformation, intermolecular interactions, and thermal gelling formation of EWPs (Liu et al., 2020). Previous evidence showed that disruption of glycosidic bonds in ovomucin triggered its conformational changes by decreasing particle size and increasing surface charge (Shan et al., 2020). Moreover, it has been established that N-glycans attached to mucin-5B undergo covalent crosslinking during the process of spray drying. This, in turn, has been shown to have an effect on the thermal aggregation behavior of EWPs (Liu et al., 2021). Comprising roughly 10% of total EWPs, ovomucoid (OVM) represents a glycosylated globulin containing sulfated and sialylated oligosaccharides (Yao et al., 2018). Its three tandem structural domains, connected via internal disulfide bonds, endow it with strong thermal stability (Chen et al., 2023). Removal of glycan moieties from OVM increased α-helix content while decreasing random coil in its secondary structure (Ma et al., 2021). The glycan structure of OVM not only influences its allergenicity and conformation but is also closely related to the gelling properties of egg white (Geng et al., 2017). Previous studies have demonstrated that when OVM complexes with ovalbumin or β-lactoglobulin, it promotes structural unfolding and thermal polymerization during heat treatment, accelerating protein thermal aggregation and facilitating egg white thermogel formation (Liu, Ma, et al., 2022). OVT, the second most abundant protein in egg white, is a glycoprotein composed of a single-chain polypeptide folded into two globular lobes (C- and N-terminal) and stabilized by 15 disulfide bonds. The C-terminal domain carried a glycan chain consisting of *N*-acetylglucosamine and mannose (Chen et al., 2023; Tong et al., 2012). Notably, the complex N-linked glycan structure of OVT can enhance steric hindrance, thereby reinforcing the three-dimensional conformation and improving the thermal stability of OVT (Liu, Wang, et al., 2022). Secondary structure analysis revealed that OVT contained α-helices, β-sheets, β-turns, and random coils, with deglycosylation significantly reducing its α-helix content (Hwang et al., 2014; Liu, Wang, et al., 2022). Additionally, the glycation preference for ovotransferrin at lysine residues may contribute to the regulation of gel structure by modifying the ratio of fibrous to granular aggregates (Yang et al., 2021). Therefore, we speculated that HF enhanced egg white protein thermal stability through decreasing the levels of Hex_4_HexNAc_6_ and Hex_3_HexNAc_6_ on Mucin-5B (N69), as well as Hex_4_HexNAc_3_NeuAc1, Hex_3_HexNAc_7_, and Hex_5_HexNAc_6_ on OVM (N199 and N190), while increasing the abundance of Hex_9_GlcNAc_2_, Hex_3_HexNAc_7_, and Hex_4_HexNAc_4_ modifications on OVT (N492). These changes can promote a transition from disordered to ordered secondary structures in EWPs, thereby reducing the rate of protein thermal aggregation and improving egg white thermal gel characteristics.

Random coils were disordered, while α-helices and β-sheets represented ordered secondary structures (Li, Guan, et al., 2025). Meanwhile, α-helices and β-sheets in the secondary structure were found to be stabilized with abundant hydrogen bonds, while random coil and β-turn were found to lack such stable hydrogen-bonding networks (Ding et al., 2020). Thus, this research discovered that HF increased β-sheets, α-helices, and hydrogen bonds in thermal gels, indicating that HF may stabilize the α-helix and β-sheet in the secondary structure of egg white thermogels by increasing hydrogen bonds, thereby making the thermally induced egg white gel structure more ordered in late-laying hens. Furthermore, increased β-sheet content has been shown to improve egg white gel hardness by inducing protein unfolding, which in turn facilitates intermolecular cross-linking and the formation of a denser gel (Chen et al., 2024; Shen et al., 2017). Shan et al. (2020) also found that the decline in the HU correlated directly with ovomucin glycosidic bond cleavage, which caused the secondary structure of ovomucin to transform from β-sheets and α-helices into random coils, thereby reducing its thermally induced gelling characteristics. Separately, N-glycosylation positioned between ordered secondary structural segments can govern protein folding and stability by means of steric hindrance and local conformational preferences (Chen et al., 2010).

Hence, we further examined changes in egg white protein tertiary conformation using intrinsic fluorescence spectroscopy. Generally, an increase in fluorescence intensity indicates that aromatic residues are more exposed on the protein surface, reflecting an unfolding or extension of its three-dimensional structure, whereas a decrease in fluorescence intensity suggests that the residues are buried within a more compact hydrophobic core (Wang et al., 2024). As reported, the fluorescence intensity gradually increased with rising aspartic acid concentration, which was attributed to protein unfolding (Zang et al., 2025a). Similarly, the increase of glycosylation modifications in pigskin collagen led to the unfolding of its tertiary structure, which helped prevent the formation of large aggregates in the gel, thereby forming a dense gel network structure (Jiang et al., 2025). In the present study, HF significantly increased the intrinsic fluorescence intensity in egg white thermogels, suggesting that HF unfolded tertiary structures in egg white thermogels. Taken together, our results demonstrated that HF modulated protein site-specific N-glycan structural features, shifting secondary structure from disordered to ordered and unfolding tertiary structure, thereby ameliorating thermal gelling characteristics of EWPs in late-laying hens.

## 5 Conclusions

Here, we provided a comprehensive egg white protein N-glycoproteomic atlas, achieving site-resolved N-glycan structural features through intact glycopeptide analysis, which enabled the identification of 767 unique N-glycopeptides modified by 76 distinct N-glycans across 46 N-glycoproteins. Meanwhile, we systematically delineated the macro- and microheterogeneity of N-glycosylation in egg white glycoproteins across glycosites, glycoproteins, glycoforms, and co-occurring glycans, a heterogeneity that can manifest in numerous forms, including variations among N-glycosites on the same protein. Co-occurrence network analysis further revealed microheterogeneity among N-glycans attached to the same glycosites, along with a preferential co-occurrence of structural features within individual glycans. Furthermore, comparison of site-specific N-glycans across different N-glycan categories revealed that HF promoted egg white protein stability by increasing paucimannose and sialylated N-glycans, and also reduced the abundance of Hex_4_HexNAc_6_ and Hex_3_HexNAc_6_ on mucin-5B N69, as well as Hex_4_HexNAc_3_NeuAc_1_, Hex_3_HexNAc_7_, and Hex_5_HexNAc_6_ on ovomucoid N199 and N190, while increasing the abundance of Hex_9_GlcNAc_2_, Hex_3_HexNAc_7_, and Hex_4_HexNAc_4_ modifications on OVT N492. These changes promoted the formation of more ionic bonds, hydrophobic interactions, and hydrogen bonds, thereby converting the secondary structure from disordered to ordered and ultimately improving egg white gel properties. In conclusion, the research establishes the site-specific N-glycosylation landscape of EWPs and provides a novel perspective for investigating the improvement of gel properties through the regulation of structural and site-specific N-glycans of EWPs.

## CRediT authorship contribution statement

**Guang Li:** Writing – review & editing, Writing – original draft, Supervision, Project administration, Investigation, Funding acquisition, Data curation. **Jiao Wang:** Validation, Data curation. **Pengyun Gong:** Methodology, Data curation. **Yongguo Jin:** Supervision, Data curation. **Bingkun Zhang:** Validation, Investigation. **Yuming Guo:** Supervision, Conceptualization. **Zengpeng Lv:** Writing – review & editing, Supervision, Funding acquisition, Conceptualization.

## Ethics declaration

### Animal subject

This study was conducted in accordance with the ARRIVE (Animal Research: Reporting of In Vivo Experiments) guidelines.

This study was approved by the the Animal Care and Use Committee of China Agricultural University. (Approval No. AW02105202–1-1).

## Declaration of competing interest

The authors declare that they have no known competing financial interests or personal relationships that could have appeared to influence the work reported in this paper.

## Data Availability

All data generated or analyzed during this study are available from the corresponding author on reasonable request.
