# Supplementary material for: Site-specific N-glycoproteomics reveals alteration of N-glycan structures and heterogeneity to enhance thermally induced gelation characteristics of egg white proteins
Source: Food Chem X. 2026 Sep 20;39:104473. doi: 10.1016/j.fochx.2026.104473 (PMC13631889; doi:10.1016/j.fochx.2026.104473)
Supplement: Supplementary file 1 [file mmc1.docx]

Fig. S1. (a-b) Examples of the MS/MS spectra of the intact N-glycopeptides: Ovomucoid (P01005) with “Hex_10_HexNAc_2_” (a) and Ig lambda chain C region (P20763) with “Hex_5_HexNAc_4_FucNeuAc” (b).
