# Supplementary material for: Site-specific N-glycoproteomics reveals alteration of N-glycan structures and heterogeneity to enhance thermally induced gelation characteristics of egg white proteins
Source: Food Chem X. 2026 Sep 20;39:104473. doi: 10.1016/j.fochx.2026.104473 (PMC13631889; doi:10.1016/j.fochx.2026.104473)
Supplement: Supplementary file 2 [file mmc2.docx]

**Table S1. Changes in T2 relaxation parameters of egg white gel**

|  | Control | HF |
| --- | --- | --- |
| T_21_ | 0.427 ± 0.026 | 0.360 ± 0.025 |
| T_22_ | 83.896 ± 1.319^a^ | 74.320 ± 1.336^b^ |
| T_23_ | 1301.573 ± 65.908^a^ | 2066.036 ± 282.627^b^ |
| PT_21_% | 1.056 ± 0.091 | 0.911 ± 0.034 |
| PT_22_% | 97.984 ± 0.193^b^ | 98.731 ± 0.164^a^ |
| PT_23_% | 0.960 ± 0.117^a^ | 0.358 ± 0.054^b^ |

Notes: Without a common letter in the figure represent significant differences at the level of *p* < 0.05.

**Table S2. Glycosites occupied by over five site-specific N-glycans between control and HF groups**

| **Uniprot accession no.** | **Protein** | **Peptide sequence** | **Total number (Control/HF) ^a^** | **High mannose (Control/HF)** | **Paucimannose (Control/HF)** | **Fucosylated-only (Control/HF)** | **Sialylated-only (Control/HF)** | **Sialofucosylated (Control/HF)** | **Undecorated (Control/HF)** |
| --- | --- | --- | --- | --- | --- | --- | --- | --- | --- |
| F1NBL0 | Mucin-6 | SSSLPH^387^NGTLMAIYEK | 4\|4 | 1\|1 | 0\|0 | 1\|1 | 0\|0 | 0\|0 | 2\|2 |
| F1NBL0 | Mucin-6 | YVNIEGCY^1133^NCSHDEYFDYEKEK | 6\|1 | 1\|0 | 0\|0 | 3\|1 | 0\|0 | 0\|0 | 2\|0 |
| F1NBL0 | Mucin-6 | ^223^NCTCSTLSEYSR | 2\|2 | 0\|0 | 0\|0 | 0\|0 | 0\|0 | 0\|0 | 2\|2 |
| F1NBL0 | Mucin-6 | TCSNPEYSCSSHCTYGCFCPEGTVLDDISK^303^NR | 1\|1 | 0\|0 | 0\|0 | 1\|1 | 0\|0 | 0\|0 | 0\|0 |
| P01005 | Ovomucoid | FP^34^NATDKEGK | 57\|57 | 7\|7 | 9\|9 | 7\|6 | 8\|9 | 1\|1 | 25\|25 |
| P01005 | Ovomucoid | EHDGECKETVPM^93^NCSSYAJTTSEDGK | 35\|34 | 3\|3 | 4\|3 | 3\|3 | 2\|2 | 0\|0 | 23\|23 |
| P01005 | Ovomucoid | EHDGECKETVPMJCSSYA^99^NTTSEDGK | 39\|39 | 5\|5 | 6\|6 | 2\|2 | 1\|1 | 0\|0 | 25\|25 |
| P01005 | Ovomucoid | C^190^NFCNAVVESJGTLTLSHFGK | 32\|26 | 1\|0 | 3\|3 | 4\|1 | 5\|3 | 0\|0 | 19\|19 |
| P01005 | Ovomucoid | CJFCNAVVES^199^NGTLTLSHFGKC | 34\|31 | 2\|2 | 4\|4 | 3\|3 | 5\|3 | 0\|0 | 20\|19 |
| P01005 | Ovomucoid | DLRPICGTDGVTYTJDCLLCAYSIEFGT^77^NISK | 22\|13 | 2\|1 | 3\|2 | 0\|0 | 1\|0 | 0\|0 | 16\|10 |
| P01005 | Ovomucoid | AFNPVCGTDGVTYD^128^NECLLCAHKVEQGASVDKR | 3\|3 | 0\|0 | 1\|1 | 1\|1 | 0\|0 | 0\|0 | 1\|1 |
| P01005 | Ovomucoid | ELAAVSVDCSEYPKPDCTAEDRPLCGSD^182^NKTYGJK | 5\|6 | 0\|0 | 0\|2 | 0\|0 | 0\|0 | 0\|0 | 5\|4 |
| P01005 | Ovomucoid | TYG^187^NKCJFCNAVVESJGTLTLSHFGK | 1\|1 | 0\|0 | 0\|0 | 0\|0 | 0\|0 | 0\|0 | 1\|1 |
| P01012 | Ovalbumin | MEEKY^293^NLTSVLMAMGITDVFSSSAJLSGISSAESLK | 9\|4 | 4\|2 | 0\|0 | 0\|0 | 0\|0 | 0\|0 | 5\|2 |
| P01012 | Ovalbumin | MEEKYJLTSVLMAMGITDVFSSSA^312^NLSGISSAESLK | 1\|0 | 0\|0 | 0\|0 | 0\|0 | 0\|0 | 0\|0 | 1\|0 |
| P01013 | Ovalbumin-related protein X | QESKPVQMMCM^59^NNSFNVATLPAEK | 22\|23 | 1\|1 | 3\|3 | 1\|1 | 3\|4 | 0\|0 | 14\|14 |
| P01014 | Ovalbumin-related protein Y | EMPFSMTKEESKPVQMMCM^215^NNSFNVATLPAEK | 25\|25 | 1\|1 | 4\|4 | 1\|1 | 3\|4 | 0\|0 | 16\|15 |
| P01014 | Ovalbumin-related protein Y | ELLSEITRP^95^NATYSLEIADK | 6\|6 | 1\|1 | 1\|1 | 0\|0 | 0\|0 | 0\|0 | 4\|4 |
| P01014 | Ovalbumin-related protein Y | MKIEEKY^293^NLTSILMALGMTDLFSR | 7\|1 | 0\|0 | 0\|0 | 0\|0 | 0\|0 | 0\|0 | 7\|1 |
| P02752 | Riboflavin-binding protein | CVPYSEMYA^164^NGTDMCQSMWGESFK | 11\|8 | 1\|0 | 0\|0 | 0\|0 | 0\|0 | 0\|0 | 10\|8 |
| P02752 | Riboflavin-binding protein | VSNSYW^72^NR | 1\|1 | 0\|0 | 0\|0 | 0\|0 | 0\|0 | 0\|0 | 1\|1 |
| P02789 | Ovotransferrin | TAGWVIPMGLIH^492^NR | 21\|24 | 6\|7 | 2\|4 | 0\|0 | 0\|0 | 0\|0 | 13\|13 |
| P02789 | Ovotransferrin | RFGV^637^NGSEKSK | 3\|3 | 0\|0 | 0\|0 | 0\|0 | 0\|0 | 0\|0 | 3\|3 |
| P10184 | Ovoinhibitor | ILKPVCGSDSFTYDJECGICAYNAEHHT^186^NISK | 19\|19 | 1\|1 | 1\|1 | 2\|2 | 1\|1 | 0\|0 | 14\|14 |
| P10184 | Ovoinhibitor | CFFCNAYVQS^506^NR | 17\|19 | 3\|3 | 2\|2 | 0\|1 | 2\|2 | 0\|0 | 10\|11 |
| P10184 | Ovoinhibitor | ILKPVCGSDSFTYD^172^NECGICAYNAEHHTJISK | 1\|1 | 0\|0 | 0\|0 | 0\|0 | 0\|0 | 0\|0 | 1\|1 |
| P20740 | Ovostatin | VIQLFVNNKNTH^403^NFTTDINGIAPFSIDTSK | 6\|5 | 3\|2 | 1\|1 | 0\|0 | 0\|0 | 0\|0 | 2\|2 |
| P20740 | Ovostatin | VVLEYDTI^82^NTTIFEK | 7\|6 | 2\|2 | 0\|0 | 0\|0 | 0\|0 | 0\|0 | 5\|4 |
| P20740 | Ovostatin | YNIHLPEGAFGFSLSVQTS^1347^NASCPR | 1\|1 | 0\|0 | 0\|0 | 0\|0 | 0\|0 | 0\|0 | 1\|1 |
| P20763 | Ig lambda chain C region | VTH^90^NGTSITK | 12\|12 | 0\|0 | 0\|0 | 6\|6 | 1\|1 | 2\|2 | 3\|3 |
| Q5ZJ97 | Uncharacterized protein KIAA1143 homolog | VLDF^117^NVSSSK | 5\|5 | 0\|0 | 0\|0 | 0\|0 | 0\|0 | 0\|0 | 5\|5 |
| Q90839 | Dickkopf-related protein 3 | TVQTHQEIDKVTD^113^NR | 14\|15 | 0\|0 | 0\|1 | 0\|0 | 0\|0 | 0\|0 | 14\|14 |
| Q90839 | Dickkopf-related protein 3 | KLSEVNFENLPPTYH^88^NESNTETR | 3\|3 | 0\|0 | 0\|0 | 0\|0 | 0\|0 | 0\|0 | 3\|3 |
| Q98UI9 | Mucin-5B | VYSSGGTYSTPCQ^381^NCTCK | 16\|16 | 3\|3 | 3\|3 | 1\|1 | 0\|0 | 0\|0 | 9\|9 |
| Q98UI9 | Mucin-5B | VQMKPVMQLSITVDHSYQ^528^NR | 19\|18 | 7\|7 | 4\|4 | 0\|0 | 0\|0 | 0\|0 | 8\|7 |
| Q98UI9 | Mucin-5B | YC^680^NQSCR | 15\|14 | 4\|3 | 2\|2 | 0\|0 | 0\|0 | 0\|0 | 9\|9 |
| Q98UI9 | Mucin-5B | RQW^855^NCTDNPCK | 16\|16 | 1\|1 | 2\|2 | 0\|0 | 0\|0 | 0\|0 | 13\|13 |
| Q98UI9 | Mucin-5B | TCII^1841^NK | 10\|10 | 1\|1 | 1\|1 | 0\|0 | 0\|0 | 0\|0 | 8\|8 |
| Q98UI9 | Mucin-5B | TYPL^1219^NETIYSQTEGTK | 10\|10 | 1\|1 | 0\|0 | 0\|0 | 0\|0 | 0\|0 | 9\|9 |
| Q98UI9 | Mucin-5B | E^1452^NCTYVLVELIQPSSEK | 1\|1 | 1\|1 | 0\|0 | 0\|0 | 0\|0 | 0\|0 | 0\|0 |
| Q98UI9 | Mucin-5B | SPPI^1807^NCTK | 18\|19 | 0\|0 | 4\|4 | 0\|0 | 0\|0 | 0\|0 | 14\|15 |
| Q98UI9 | Mucin-5B | FTFPGTCTYVFASHC^69^NDSYQDFNIK | 3\|0 | 0\|0 | 0\|0 | 0\|0 | 0\|0 | 0\|0 | 3\|0 |
| Q98UI9 | Mucin-5B | FAQHWCALLS^599^NTSSTFAACHSVVDPSVYIKR | 3\|1 | 0\|0 | 0\|0 | 0\|0 | 0\|0 | 0\|0 | 3\|1 |
| Q98UI9 | Mucin-5B | QGICDPSEECPETMVY^673^NYSVK | 1\|1 | 1\|1 | 0\|0 | 0\|0 | 0\|0 | 0\|0 | 0\|0 |
| Q98UI9 | Mucin-5B | ITSTCSNI^1036^NMTDLCADQPFK | 1\|1 | 0\|0 | 0\|0 | 0\|0 | 0\|0 | 0\|0 | 1\|1 |
| Q98UI9 | Mucin-5B | KF^1371^NESWDFGNCQIATCLGEENNIK | 10\|6 | 0\|0 | 0\|0 | 0\|0 | 0\|0 | 0\|0 | 10\|6 |
| Q98UI9 | Mucin-5B | YY^1567^NNTMGLCGTCTNQK | 4\|4 | 0\|0 | 0\|0 | 0\|0 | 0\|0 | 0\|0 | 4\|4 |
| Q98UI9 | Mucin-5B | IIW^1639^NLTECHR | 4\|4 | 0\|0 | 0\|0 | 0\|0 | 0\|0 | 0\|0 | 4\|4 |
| Q98UI9 | Mucin-5B | R^1716^NTCFSR | 1\|1 | 1\|1 | 0\|0 | 0\|0 | 0\|0 | 0\|0 | 0\|0 |
| Q98UI9 | Mucin-5B | EAWEHDCQYCTCDEETL^1792^NISCFPRPCAK | 7\|6 | 0\|0 | 0\|0 | 0\|0 | 0\|0 | 0\|0 | 7\|6 |
| Q98UI9 | Mucin-5B | SYKAPYD^1964^NCTQYTCTESGGQFSLTSTVK | 1\|0 | 0\|0 | 0\|0 | 0\|0 | 0\|0 | 0\|0 | 1\|0 |

^a^ Control/HF represents numbers of intact N-glycopeptides identified in egg white glycoproteins from the Control and HF groups, respectively.
